# Two Clusters of HIV-1 Infection, Rural Idaho, USA, 2008

**DOI:** 10.3201/eid1611.100857

**Published:** 2010-11

**Authors:** Randall J. Nett, Jared L. Bartschi, Giovanina M. Ellis, David M. Hachey, Lisa M. Frenkel, J. Clay Roscoe, Kris K. Carter, Christine G. Hahn

**Affiliations:** Author affiliations: Centers for Disease Control and Prevention, Atlanta, Georgia, USA (R.J. Nett, K.K. Carter);; Idaho Department of Health and Welfare, Boise, Idaho, USA (R.J. Nett, J.L. Bartschi, J.C. Roscoe, K.K. Carter, C.G. Hahn);; Seattle Children’s Hospital Research Institute, Seattle, Washington, USA (G.M. Ellis, L.M. Frenkel);; Idaho State University, Pocatello, Idaho, USA (D.M. Hachey);; University of Washington, Seattle (L.M. Frenkel);; Family Medicine Residency of Idaho, Boise (J.C. Roscoe)

**Keywords:** Viruses, HIV, epidemiology, rural population, Idaho, men who have sex with men, MSM, intravenous substance abuse, infectious disease transmission, Idaho, letter

**To the Editor:** Prevalence of HIV-1 infection in rural areas of the United States has been increasing (*1*). During 2003–2007, an average of 30 (range 24–42) cases of new HIV-1 infection diagnoses per year among Idaho residents were reported. Of the 152 reported cases during this period, 54 (36%) were related to a person living in a rural area of <75,000 residents and a 60-minute drive from an urban area (*2*). Of these 54 cases, 19 (35%) were in men who have sex with men (MSM), 5 (9%) were in injection drug users (IDU), and 2 (4%) were in those in both categories.

In March 2008, a cluster of newly identified HIV-1 infections that included 5 cases (cluster A) in a rural southeastern Idaho city (city A) was reported to the Idaho Department of Health and Welfare. Two patients were men and the median age was 26 years (range 18–32 years). One patient was an IDU (Table). Through epidemiologic investigation, 3 additional patients were suspected to be IDUs, but confirmation was not practicable. All reported methamphetamine use. One man and 2 women reported both male and female sex partners.

**Table:** Sex and risk factors among patients epidemiologically linked to 2 clusters of  HIV-1, southeastern Idaho, USA, 2008*

Case ID	Sex	Risk factors	Cluster†	Phylogenetic group
1	F	WSMW/SIDU	A	1
2	F	WSMW/SIDU	A	1
3	M	MSMW/IDU	A	1
4‡	M	MSW	A	ND
5	M	MSM	B	2
6‡	M	MSM	B	ND
7‡	M	MSM	B	ND
8‡	M	MSM	B	ND
9	M	MSM	B	2
10§	M	MSM	B	ND
11	M	MSM	B	2
12‡	M	MSM	B	ND
13	M	MSM	B	2
14	F	WSM	A	1
15	M	MSM	B	2
16¶	M	MSM	ND	1

*ID, identification; WSMW, women who have sex with men and women; SIDU, sex with injection drug users; MSMW, men who have sex with men and women; IDU, injection drug user; MSW, men who have sex with women; ND, not determined; MSM, men who have sex with men; WSM, women who have sex with men. †Cluster A associated with injection drug use; cluster B associated with MSM. ‡HIV-1 sequence was not available for molecular analysis. §HIV-1 sequence was similar to controls but not to sequences from either cluster A or B. ¶Case with no epidemiologic link to cluster A or cluster B.

During September–December of that year, another increase in newly identified HIV-1 infections in southeastern Idaho (cluster B) was reported to Idaho Department of Health and Welfare. Cluster B included 10 cases, all among men who reported living within a 50-mile radius of city A, with most in a rural city (city B) located <30 miles from city A. The median age of the men in cluster B was 24 years (range 18–37 years). Each case was epidemiologically linked to at least 1 other case in the cluster; each patient reported having had unprotected sex with male partners. Although we suspected transmission of HIV-1 between persons in clusters A and B, whether the clusters were linked epidemiologically remained unclear after an initial investigation.

Although the primary use of HIV-1 sequence data is to assist clinicians in selecting antiretroviral (ARV) therapy, public health practitioners can use HIV-1 sequences from cases and compare those with HIV-1 sequences from others living in the region to explore phylogenetic associations and possible HIV transmission clusters (*3*). To evaluate links between clusters A and B, HIV-1 *pol* consensus sequence data for 4 of the 5 cases from cluster A and 6 of the 10 cases from cluster B were obtained from 5 commercial laboratories. No case-patients had received ARV. Additionally, we used sequence data from a patient residing in city B who had received an HIV-1 diagnosis in December 2008 but was not epidemiologically linked to either cluster. HIV-1 control sequences from 2 Idaho HIV clinics, including 34 HIV-1–infected persons within a 275-mile radius of city B identified who had not received ARV and who had resistance testing performed during 2005–2008, were used to represent the regional epidemic. Control sequences were aligned with cluster A and B sequences and analyzed as described (*4*,*5*).

Ten of the HIV case-patients for whom nucleotide sequence data were obtained were infected with HIV-1, subtype B, and were placed into 2 distinct phylogenetic-related groupings. Group 1 contained 4 patients from cluster A and 1 patient with no known epidemiologic link to either cluster. Group 2 contained 5 patients from cluster B. The average *pol* genetic distance among virus from members of group 1 was 0.2% (median 0.1%, SD 0.2%) and from members of group 2 was 0.1% (median 0.1%, SD 0.1%). The average distance among the control sequences was 5.1% (median 5.2%, SD 1.2%). The average distance between groups 1 and 2 was 4.8% (median 4.8%, SD 0.2%), which does not demonstrate a linkage between the 2 groups. The 1 case in group 1 that was not initially identified with either cluster had a genetically related HIV-1 sequence to members of cluster A, indicating a potential previously unidentified epidemiologic link. The sequence from 1 case associated with cluster B was not genetically similar to members of either cluster and was more similar to controls. These data do not indicate from whom each patient acquired the infection.

The epidemiologic investigation combined with the molecular analysis shows transmission of HIV-1 originating from 2 sources occurred within a group of rural MSM in southeastern Idaho and indicates a separate case previously believed to be unrelated to 2 local clusters had genetic similarity to cluster A. Limitations of this investigation include the inability to obtain HIV sequences from all persons identified in clusters A and B and an inability to confirm high-risk behaviors for all identified case-patients.

Previous HIV clusters have demonstrated that infectious persons can spread HIV quickly within a social network and highlighted the importance of timely prevention activities to limit HIV transmission in a community (*6*,*7*). Use of phylogenetic analysis of HIV-1 sequences obtained from commercial laboratories showed that clusters A and B were not epidemiologically related and helped target appropriate and specific HIV prevention activities.

## References

[1] Hall HI, Li J, McKenna MT. HIV in predominantly rural areas of the United States. J Rural Health. 2005;21:245–53. 10.1111/j.1748-0361.2005.tb00090.x 16092299

[2] Bowen A, Williams M, Horvath K. Using the Internet to recruit rural MSM for HIV risk assessment: sampling issues. AIDS Behav. 2004;8:311–9. 10.1023/B:AIBE.0000044078.43476.1f 15475678 PMC2614667

[3] Robbins KE, Weidle PJ, Brown TM, Saekhou AM, Coles B, Holmberg SD, Molecular analysis in support of an investigation of a cluster of HIV-1–infected women. AIDS Res Hum Retroviruses. 2002;18:1157–61. 10.1089/088922202320567914 12402955

[4] Thompson JD, Gibson TJ, Plewniak F, Jeanmougin F, Higgins DG. The CLUSTAL_X windows interface: flexible strategies for multiple sequence alignment aided by quality analysis tools. Nucleic Acids Res. 1997;25:4876–82. 10.1093/nar/25.24.4876 9396791 PMC147148

[5] Buskin SE, Ellis GM, Pepper GG, Frenkel LM, Pergam SA, Gottlieb GS, Transmission cluster of multiclass highly drug-resistant HIV-1 among 9 men who have sex with men in Seattle/King County, WA, 2005–2007. J Acquir Immune Defic Syndr. 2008;49:205–11. 10.1097/QAI.0b013e318185727e 18769347 PMC2586929

[6] Centers for Disease Control and Prevention. Cluster of HIV-positive young women—New York, 1997–1998. MMWR Morb Mortal Wkly Rep. 1999;48:413–6. 10365630

[7] Denoon DJ. CDC warns of HIV “clusters” in low-prevalence areas. AIDS Wkly Plus. 1999;19:3–4. 12295183

